# Prospect of Wearable Ultrasound

**DOI:** 10.34133/bmef.0271

**Published:** 2026-05-18

**Authors:** Yushun Zeng, Qifa Zhou

**Affiliations:** ^1^ Alfred E. Mann Department of Biomedical Engineering, Viterbi School of Engineering, University of Southern California, Los Angeles, CA, USA.; ^2^ USC Roski Eye Institute, Keck School of Medicine, University of Southern California, Los Angeles, CA, USA.

## Abstract

Wearable ultrasound presents a promising technological solution for continuous, noninvasive monitoring of deep-tissue structures and physiological functions, addressing the key limitation of traditional wearable sensors that are largely confined to superficial signal detection. In recent years, wearable ultrasound transducers have advanced from early flexible and stretchable array designs to high-performance platforms that combine rigid functional components with flexible architectures. This hybrid design enables more stable coupling with the body surface and supports multimodal imaging capabilities. These developments highlight the strong potential of wearable ultrasound for long-term physiological monitoring and personalized medicine. However, several critical challenges remain before fully continuous and practical applications can be realized. This perspective reviews recent progress in wearable ultrasound technology and discusses future directions, such as the development of compact wireless systems, the incorporation of artificial intelligence, the expansion to 2-dimensional array configurations, and the creation of multimodal functional platforms.

## Background of Wearable Ultrasound

Recently, the rapid advancement of flexible and wearable electronic devices has been reshaping the way that humans approach health monitoring and medical diagnosis [1,2]. Wearable devices represented by smartwatches, flexible physiological sensors, and continuous vital sign monitoring systems have facilitated a gradual shift from traditional hospital settings to daily life environments, enabling the continuous, real-time, noninvasive, conformal, and long-term monitoring of human physiological states [3–6]. However, the function of the most existing wearable devices primarily relies on monitoring the electrical signals or superficial physiological parameters, including heart rate, body temperature, blood oxygen, or skin electrical signals, and the capacity to acquire structural information regarding deep tissues and internal organs remains limited.

Given its unique background, wearable ultrasound technology has gradually emerged as a new research direction in biomedical engineering [7]. As a modality that is biosafe, noninvasive, radiation-free, relatively low-cost, and capable of real-time imaging, ultrasound has played an important role in clinical diagnosis and treatment [8,9]. However, the current ultrasound-related research is fundamentally limited by the ultrasound transducers, devices that generate and transmit the ultrasound. Consequently, developing newly advanced wearable ultrasound transducers has recently attracted lots of attention from biomedical researchers. Integrating ultrasound transducers with wearable systems enables the continuous monitoring of deep tissue structures and functions, thereby offering new technical pathways for chronic disease management, organ function assessment, and dynamic physiological studies [10]. Compared to traditional, bulky ultrasound transducers, wearable ultrasound transducers possess several potential advantages, including continuous monitoring, user-friendliness, and allowing the free movement of the patients. These characteristics position wearable ultrasound to drive the transition of medical imaging from episodic diagnosis to continuous and long-term health monitoring while also serving as an essential tool for the future of personalized medicine.

In this perspective, we aim to not only provide a comprehensive overview of current wearable ultrasound technologies but also demonstrate a framework that connects system engineering, artificial intelligence (AI), device structures, and multifunction integration in next-generation wearable ultrasound. We highlight wireless and highly integrated system architectures for real-world applications and discuss how AI can fundamentally reshape medical ultrasound diagnosis and interpretation. Furthermore, we point out future 2-dimensional (2D) ultrasound array transducers and multi-functions, and potential clinical use as critical directions for achieving imaging and therapeutic functions. We anticipate that such an integrated perspective will accelerate the transition of wearable ultrasound from laboratory prototypes to practical healthcare solutions.

## Progress and Limitations of Current Wearable Ultrasound

A series of pioneering research efforts drove the advancement of flexible and wearable ultrasound technology. For instance, a stretchable ultrasonic transducer array was reported by integrating piezoelectric elements into an elastic substrate using an island-bridge architecture [11]. Developed ultrasound array transducer was capable of conforming to complex curved surfaces, thereby enabling potential ultrasound imaging of nonplanar structures, demonstrating the feasibility of integrating ultrasound arrays onto a stretchable electronics platform. Subsequently, an ultrasound device was further developed to be capable of conformal contact with human skin, utilizing for the continuous monitoring of central blood pressure waveforms and thereby demonstrating the significant potential of wearable ultrasound for continuous physiological monitoring [12]. These studies validated the feasibility of flexible and wearable ultrasound devices for long-term attachment to the human body and for continuous monitoring. Nevertheless, early flexible and wearable ultrasound transducers still faced significant limitations. Constrained by device architecture and packaging methods, their operating frequencies were typically low (<10 MHz), making it difficult to meet the bandwidth and array performance requirements essential for high-resolution and high-precision medical imaging. Furthermore, these devices often lacked stable array structures and precise signal control capabilities, thereby hindering the implementation of advanced ultrasound imaging algorithms, such as high-resolution B-mode imaging or functional imaging algorithms. Consequently, while early flexible ultrasound transducers held significant conceptual value for proof-of-concept demonstrations, they remained markedly inadequate for high-quality continuous medical imaging applications.

## Future Directions of Wearable Ultrasound

To enhance the imaging performance and system stability of wearable ultrasound transducers, researchers have recently proposed a series of novel structural design strategies. These strategies involve the systematic integration of high-performance ultrasound transducer arrays with mechanically compliant wearable structures, thereby enabling long-term, stable adhesion to the human body while simultaneously ensuring high imaging quality. A representative example of this approach is the wearable ultrasound array transducer based on a printed circuit board (PCB)-belt structure [13]. This design utilized a hybrid structure combining rigid and linear piezoelectrical active array areas with flexible PCB-belt substrate, which ensured transducer efficiency, array performance uniformity, and signal integrity. The belt structures can conform to the curvature variations of the human or animal body surface and achieve stable wearability. By balancing the high-performance operation of the ultrasound array with the requisite comfort and mechanical reliability of a wearable ultrasound transducer, this structural strategy offers a crucial engineering pathway for realizing long-term dynamic monitoring.

In addition, another significant ​​progress is bioadhesive coupling layers for assembling wearable ultrasound transducers. Relevant research involves designing adhesive coupling materials characterized by excellent biocompatibility and acoustic impedance matching properties, thereby establishing a stable, low-loss acoustic transmission interface between the ultrasound array and the human skin [14,15]. This adhesive interface not only significantly enhances acoustic energy transmission efficiency and signal stability but also maintains stable acoustic coupling conditions throughout prolonged periods of wearing. Simultaneously, the entire active area of the transducer is rigid and encapsulated in the flexible adhesive substrate, enabling it to conform to the complex contours of the human body while maintaining reliable contact and high-quality imaging. Based on these innovations in material and structural design, modern wearable ultrasound transducers are now capable of supporting a variety of advanced and functional ultrasonic imaging modalities, including high-resolution B-mode imaging and shear wave elastography.

**Fig. 1.**
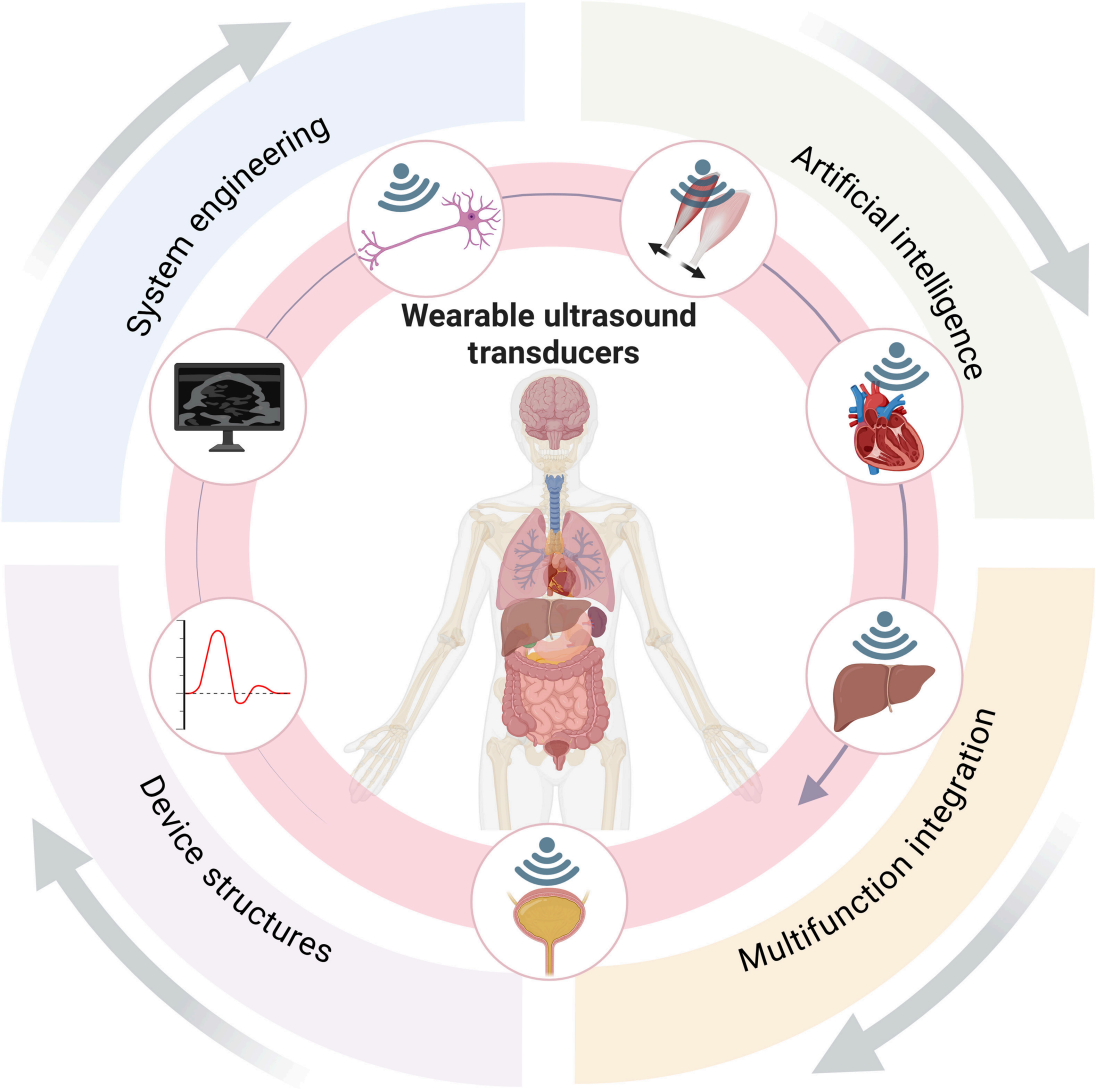
Schematic diagram of the future development in wearable ultrasound transducers.

Looking ahead, wearable ultrasound technology remains in a phase of rapid evolution. Further advancement will depend on breakthroughs across multiple dimensions, including system engineering, AI, device structures, and multifunction integration (Fig. 1). Among these, the wireless and highly integrated design of wearable systems will be a pivotal direction driving wearable ultrasound transducers toward practical real-world applications. Currently, most wearable ultrasound transducers still rely on external driving units and wired connections to handle signal excitation, data acquisition, and image processing. This driving method limits their potential for truly continuous, wearable operation. Future wearable ultrasound transducers are poised to evolve into highly integrated, miniaturized platform systems. Currently, there are few researches that introduce fully integrated ultrasound system [16–18]. By consolidating power management, high-voltage pulse driving, piezoelectric active stacks, signal reception and processing, and wireless communication modules within a single transducer device, these systems will achieve complete integration, spanning the entire process from acoustic wave generation to image reconstruction. Furthermore, through the combination of low-power electronic design, application-specific integrated circuits (ASICs), and edge computing architectures, wearable ultrasound transducers are expected to significantly reduce power consumption while maintaining high imaging performance. Moreover, by leveraging wireless communication to facilitate real-time data transmission and telemedicine connectivity with mobile terminals, future wearable ultrasound transducer system will ultimately enable truly long-term, continuous wearable ultrasound monitoring for human healthcare.

As system integration continues to advance, the deep convergence of AI and wearable ultrasound transducers will further expand the boundaries of their applications. Traditional ultrasound diagnostics rely heavily on experienced clinicians to perform probe positioning, image acquisition, and result interpretation. This dependency, to some extent, limited its widespread adoption in routine health monitoring. With the evolution of deep learning and intelligent medical imaging analysis technologies, AI has been widely applied in ultrasound fields [19,20]. Future wearable ultrasound transducers are expected to be deeply coupled with AI algorithms, enabling automated signal processing, image reconstruction, and disease and health identification. By deploying intelligent algorithms directly on the device or on mobile terminals, the system can perform real-time analysis of continuously acquired ultrasound data, automatically identifying structural anomalies or functional changes. This transforms wearable ultrasound transducer from a simple imaging tool into an intelligent health monitoring entity endowed with autonomous analytical capabilities. Under this development, users can interact with the ultrasound transducers via mobile applications to obtain real-time information regarding the structure and function of their own organs, thereby driving the advancement of patient-centric precision health management and personalized healthcare.

Beyond advancements at the system and algorithmic levels, the transducer structure will also exert a profound influence on the capabilities of wearable ultrasound technology. Currently, most wearable ultrasound devices remain based on single-element or 1D linear array configurations. Recently developed wearable 2D matrix-shaped arrays are focused on multiple hemodynamics [21,22]. Consequently, the spatial information they acquire is relatively limited, making it difficult to comprehensively capture the real 3D characteristics of complex tissue structures, restricting the precise diagnosis for clinicians. In the future, through the development of high-density 2D arrays, wearable ultrasound transducers will be able to achieve more complete spatial data acquisition, thereby enabling high-quality 3D ultrasound imaging. Meanwhile, 2D array structures facilitate the spatial manipulation of acoustic fields through precise phase control, allowing ultrasound energy to be dynamically focused and scanned within a 3D space. This capability not only significantly enhances imaging resolution and volumetric imaging efficiency but also establishes a crucial technical foundation for targeted ultrasound stimulation and precision therapy, thereby unlocking new potential applications for wearable ultrasound transducers in the functional modulation and therapeutic intervention.

Importantly, building upon technological advancements, functional-level integration will become a key trend for wearable ultrasound systems. Future wearable ultrasound devices will no longer be confined to structural imaging alone; rather, they will gradually evolve into comprehensive platforms that integrate a diverse array of imaging and therapeutic functions. In addition to traditional B-mode imaging, new-developed wearable ultrasound array will incorporate various functional modes, such as elastography, Doppler imaging, and blood flow imaging. Thereby, it will enable a comprehensive assessment of tissue structure, mechanical properties, and hemodynamic characteristics. At the same time, through the precise control of acoustic field distribution, wearable ultrasound transducer will also be capable of delivering localized ultrasound stimulation or therapy, thereby achieving a closed-loop integration of diagnosis, monitoring, and intervention within a single platform. This technological track of multifunctional integration will significantly enhance the application value of wearable ultrasound in the fields of chronic disease management, rehabilitation medicine, home healthcare, and telemedicine.

Finally, beyond technological development, future wearable ultrasound technologies hold significant promise for continuous monitoring. However, the clinical translation of wearable ultrasound requires careful consideration of practical deployment scenarios, regulatory pathways, and user adoption. From a regulatory perspective, wearable ultrasound technologies represent a convergence of hardware, software, and increasingly artificial intelligence, which complicates existing approval pathways. Ensuring safety, reliability, and stable ultrasound output under continuous use is of great importance, particularly given the need for stable skin coupling for transmit ultrasound. In addition, AI-enabled wearable ultrasound system for automated image acquisition may require separate validation and regulatory clearance. Typically, clinical translation depends on rigorous validation through large-scale studies to prove the reproducibility, diagnostic accuracy, and clinical utility compared to gold-standard imaging modalities [23,24]. Besides, wearable ultrasound systems must be user-friendly for both clinicians and potentially unprofessional patient users in home settings. Addressing these regulatory and translational challenges will require close collaboration among researchers, engineers, clinicians, and regulatory bodies. The establishment of standardized evaluation frameworks, together with clearly defined clinical use cases, will be critical for translating future advanced wearable ultrasound systems into daily clinical practice.

## Summary

To sum up, driven by continuous breakthroughs in key technologies, including system miniaturization and wireless capabilities, the integration of intelligent algorithms, advancements in array structures, and multi-functional integration, wearable ultrasound can evolve from its current status as a research prototype into a clinical or translational platform for biomedical monitoring and intervention. Compared to traditional wearable physiological sensors, wearable ultrasound offers the unique advantage of noninvasively capturing structural and functional information from deep human tissues, thereby enabling long-term, continuous monitoring of deep physiological processes. It will have the great potential to play a significant role in early disease screening, chronic disease management, and personalized medicine, ultimately facilitating the integration of continuous deep physiological sensing into routine health management.
